# Enucleation of painful blind eye for refractory intraocular lymphoma after dose-limiting chemotherapy and radiotherapy

**DOI:** 10.1007/s00277-012-1452-z

**Published:** 2012-03-28

**Authors:** Prudence Po-chee Chow, Siu Lun Ho, Wico W. Lai, Wing Y. Au

**Affiliations:** 1 Department of Medicine, Queen Mary Hospital, Hong Kong, SAR China; 2 Department of Pathology, Queen Mary Hospital, Hong Kong, SAR China; 3 Department of Ophthalmology, Queen Mary Hospital, Hong Kong, SAR China; 4 Eye Institute, University of Hong Kong, Hong Kong, SAR China; 5 University Medical Unit, Queen Mary Hospital, Pokfulam, Hong Kong

Dear Editor,

A 58-year-old man presented with bilateral, persistent steroid refractory vitritis in 2005. Repeated vitreous biopsies in 2006 and 2007 were inconclusive. In late 2008, he developed vertigo and a magnetic resonance imaging (MRI) scan showed an intra-axial infratentorial tumor with marked cerebellar edema. Brain biopsy showed diffuse large B cell lymphoma (DLCL). Systemic staging, including lumbar puncture, was negative. He was treated as combined intraocular lymphoma (IOL) and primary brain lymphoma (PBL) with chemotherapy [four courses high-dose methotrexate (MTX), rituximab, vincristine, and procarbazine] followed by whole brain radiotherapy (RT, 40 Gy) [1]. This resulted in clinical and radiological remission. Further treatment was limited by poor performance status and renal impairment.

**Fig. 1:**
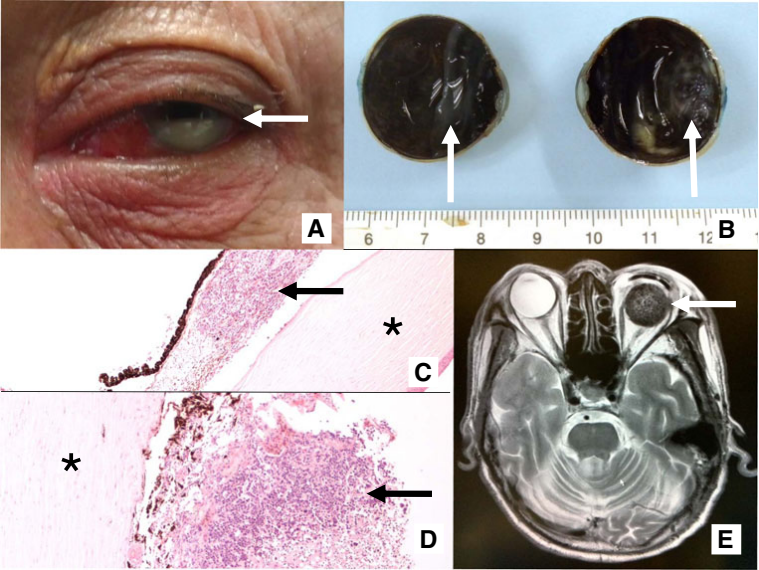
**a** Left eye hypopyon (*arrow*) of sedimentation of lymphoma cells. **b** Gross specimen bisected globe with extensive amorphous white infiltrations representing the hypopyon. **c** Photomicrograph of anterior chamber with abundant lymphoma cell infiltration (*arrows*) between the cornea (*asterisk*) and iris (×40 magnification). **d** Photomicrograph of posterior chamber with profuse lymphoma cell infiltration (*arrows*) in front of the capsule of globe (*asterisk*) and the choroid–retina layer (×40 magnification). **e** Follow-up MRI scan showing replacement glass eye (*arrow*) with no evidence of parenchymal or global disease

His vision remained impaired due to bilateral grade 3 posterior subcapsular cataract and grade 2 cortical cataract. Right eye phaco-emulsification with implantation of posterior chamber intraocular lens was performed in 2008 with good visual recovery. However, his left eye showed progressively decreased acuity to hand movement. An examination showed keratic precipitates, dense cataract, vitritis, retinal infiltrates, and hemorrhages. An MRI and lumbar puncture remained normal. He was treated as recurrent IOL with intrathecal (IT) MTX and cytosine arabinoside (Ara-C). Subsequent left eye cataract surgery and vitreous biopsy showed small lymphocytes but no malignant cells, but panuveitis persisted despite further anterior sub-Tenon’s triamcinolone acetonide injection. A repeat pars plana vitrectomy was performed in 2009. The histology showed large sized atypical lymphoid cells, but an immunogloubin heavy chain polymerase chain reaction study showed no clonality. In view of likely persistent IOL, additional dose-limiting conformal RT was given to both eyes (1.8 Gy/fr up to 28.2 Gy). Vision in his right eye remained good (0.5), but his left eye deteriorated to no light perception. By 2011, he developed left eye pain, florid vitritis, hypopyon, and glaucoma (40 mmHg, Fig. 1a). The painful blind eye was enucleated (Fig. 1b). The specimen showed abundant CD20 positive lymphoma cells in both chambers of the eye (Fig. 1c, d) with frequent mitotic figures and necrosis. Six months later, radiological and physical examinations showed no evidence of brain or orbital lymphoma (Fig. 1e).

The related pathologies of IOL and PBL may occur concurrently or sequentially. Central nervous system (CNS) investigation and therapy are advised for apparent isolated IOL [2, 3]. Meticulous fundal examination is also mandatory for PBL patients [4]. The incidence, time sequence, and interval between the entities vary and may be related to the anatomical location of the PBL lesion [5]. Histologically, both conditions are related and extra-CNS spread is uncommon [4]. The treatment includes high dose systemic and IT MTX, Ara-C, and RT [1, 3, 6]. The long-term visual, neurological, and survival prognosis is usually guarded [4, 6].

Our patient is unique in a number of ways. Although IOL is a well-known ophthalmological masquerade lesion, a 3-year lapse between IOL and PBL without treatment is unusual. In addition, the IOL was exquisitely localized to the globe, evident by its isolated nonresponse to chemotherapy, first in both eyes and eventually only in the left eye [6]. After failing multiple maximal dose therapies, the options for a frail patient become limited [1]. The hypopyon is indicative of fulminant lymphoma in the aqueous chamber [2]. Intraocular chemotherapy injection is invasive and may breach the capsular sealing of the amorphous DLCL. It is unlikely to be effective against refractory DLCL. The role of temozolamide or intraocular rituximab is uncertain [7]. Historically, the first effective treatment of IOL was enucleation (c.f. ocular melanoma and retinoblastoma) [6]. Although relegated to a historical curiosity, IOL enucleation remerged as a novel option in our very unique case with refractory localized cells in a painful blind eye. Disease and symptom control was achieved with minimal morbidity.
